# Effect of preoperative oral carbohydrate combined with postoperative early enteral nutrition on the perioperative rehabilitation of patients with colorectal cancer: a multicenter randomized controlled trial

**DOI:** 10.3389/fnut.2025.1699541

**Published:** 2025-11-28

**Authors:** Zeyin He, Yong Liu, Qiyin Xu, Xiaobo Yang, Dongbing Zhou, Lili Zhang

**Affiliations:** 1 Clinical Nutrition Section, Department of Laboratory Medicine, The Third People's Hospital of Chengdu, Chengdu, Sichuan, China; 2 Department of Nutrition and Food Hygiene, School of Public Health, Medical College of Soochow University, Suzhou, Jiangsu, China; 3 Department of Gastrointestinal Surgery, Deyang People's Hospital, Deyang, Sichuan, China; 4 Department of Gastrointestinal Surgery, The First People's Hospital of Yibin, Yibin, Sichuan, China; 5 Department of Gastrointestinal Hernia & Bariatric Metabolic Surgery, The Second People's Hospital of Yibin, Yibin, Sichuan, China; 6 Department of Gastrointestinal, Anal and Hernia Surgery, Nanchong Central Hospital, Nanchong, Sichuan, China

**Keywords:** CRC, immune function, enteral nutrition, CHOs, postoperative recovery

## Abstract

**Background:**

Preoperative oral carbohydrates (CHOs) have been widely utilized to improve perioperative outcomes. However, the effect of postoperative early enteral nutrition (EEN) intervention on patients’ postoperative recovery has yet to be validated by prospective outcomes. This study was designed to investigate the effect of preoperative oral CHOs combined with ENN nutrition on postoperative recovery in patients with colorectal cancer (CRC).

**Methods:**

A multicenter, prospective, randomized controlled study was conducted on 331 CRC patients who underwent radical resection from March 1, 2022, to March 1, 2023 and were divided into Group A (preoperative oral CHOs combined with postoperative ENN group, *n* = 110), Group B (preoperative oral CHOs group, n = 110), and Group C (conventional control group, *n* = 111) according to the method of the randomized numerical table. The general clinical data, inflammatory indices, nutrition-related serum biomarkers, immune function, postoperative intestinal function recovery, complications and hospitalization length of the three groups were statistically analyzed.

**Results:**

The baseline characteristics were similar among the groups. In the intention-to-treat analysis, the time to first exhaust (*p* < 0.05) and defecation (*p* < 0.05) was significantly shorter in group A than in groups B and C. The total protein (TP) level was significantly greater in group A than in groups B and C on the seventh postoperative day (*p* < 0.05). In addition, the percentage of T-lymphocytes to lymphocytes on the third postoperative day was greater in Group A than in Groups B and C (*p* < 0.05), and the length of hospitalization was significantly reduced. However, there was no difference in the incidence of postoperative complications.

**Conclusion:**

Preoperative oral CHOs combined with postoperative EEN improved serum markers related to postoperative nutrition, enhanced the immunity of the body, and promoted early recovery of intestinal function. Preoperative oral CHOs combined with postoperative EEN is conducive to rapid postoperative recovery and reduces the length of hospitalization.

**Clinical trial registration:**

https://www.chictr.org.cn/showproj.html?proj=144616, identifier ChiCTR2100054459.

## 1 Introduction

According to the latest statistics provided by the WHO International Centre for Research on Cancer (IARC) in 2022, the incidence of CRC is increasing in China; it is the second most prevalent malignant tumor and the fifth leading cause of death (1, 2). Although neoadjuvant therapy and targeted drugs are gradually being utilized in the treatment of colorectal patients, radical resection of CRC is still the most dominant modality for the treatment of this disease (3). Numerous studies have shown that the probability of postoperative malnutrition in oncology patients can reach 40–70%, especially in gastrointestinal malignancies and elderly patients. Malnutrition negatively affects the host immune response and tissue healing process and is an independent risk factor for postoperative complications (4). Accordingly, the risk of postoperative malnutrition is greater in CRC patients undergoing surgical resection than in those receiving other treatment modalities (5).

Enhanced recovery after surgery (ERAS) is a paradigm shift in perioperative care, using a multimodal/interdisciplinary method to care for surgical patients, improving preoperative preparation, intraoperative treatment outcomes, and early nutritional support for postoperative recovery (6). As part of the ERAS program, preoperative oral intake of CHO beverages alleviated thirst, nausea, and vomiting caused by fasting, and attenuated serum levels of postoperative inflammatory markers [cortisol, interleukin (IL)-6, IL-8, IL-10] (7–9).

Patients with CRC often experience a state of high metabolic stress following surgery, accompanied by inflammatory responses and nutritional risks. Serologically, this manifests as decreased plasma protein levels and compromised immunity (10, 11). Nutritional management during the perioperative period is crucial, and proper nutritional support accelerates postoperative patient recovery and reduces the risk of postoperative complications, thereby shortening patients’ hospital stay (12). In recent years, randomized controlled trials (RCTs) and meta-analyses have concluded that postoperative EEN reduces postoperative morbidity (especially infectious complications) (13), mortality (14), and length of hospital stay (15). However, the effect of preoperative CHOs supplementation combined with postoperative EEN on the postoperative recovery of CRC patients has not been reported.

The objective of this study was to explore the effect of preoperative CHOs supplementation combined with postoperative EEN on the postoperative recovery of patients with CRC and to provide new nutritional strategies for patients in the perioperative period.

## 2 Materials and methods

### 2.1 Participants

Participants were recruited from December 2021 to September 2022. Patients hospitalized at Deyang People’s Hospital, Yibin First People’s Hospital, Yibin Second People’s Hospital, or Nanchong Central Hospital with a diagnosis of colorectal malignancy who underwent laparoscopic CRC resection were included as study subjects. The purpose, procedures, and risks of the study were explained to each participant before inclusion, and all participants provided informed consent. The study was approved by the Ethics Committee of Deyang People’s Hospital (No. 2021–04-157-K01).

### 2.2 Study design

This study was a multicenter, double-blind, prospective, randomized controlled trial. All patients or their legal representatives signed provided written informed consent. The trial was conducted in accordance with the guidelines of the Helsinki Declaration and was registered at the Chinese Clinical Trial Registry on December 17, 2021 with registration number ChiCTR2100054459.

### 2.3 Randomization and sample size

**Figure 1:**
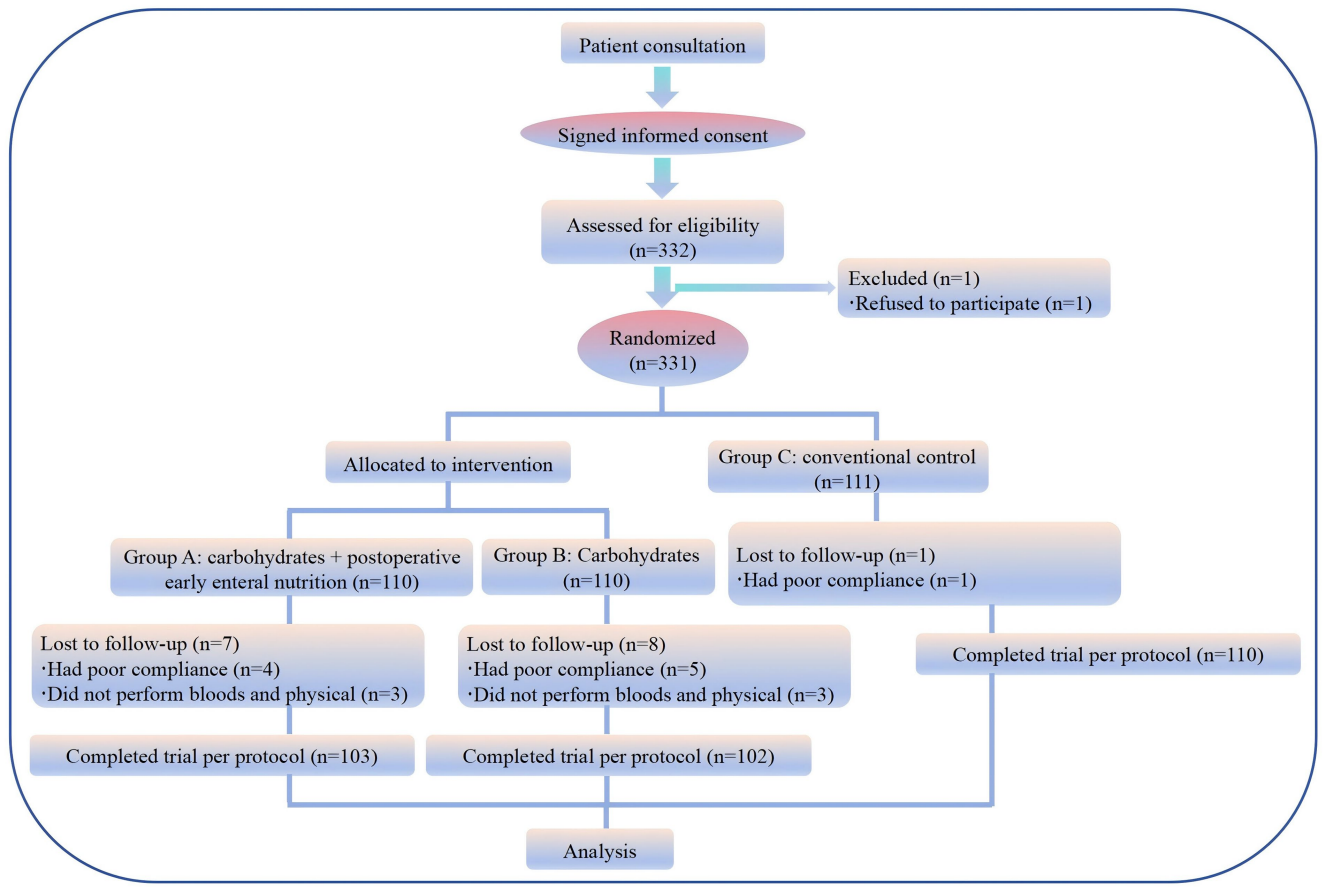
Flow diagram of the study participants. Among the study participants, 315 (94.87%) participated in this study throughout and completed the collection of baseline information and evaluation indicators.

A random number table was used to determine patient allocation. Patients were randomized (1:1:1) into a preoperative oral CHOs combined with postoperative EEN group (Group A), a preoperative oral CHOs group (Group B) and a conventional diet group (Group C). The trial parameters were set as follows: two-tailed *α* = 0.05, 1-*β* = 80%. The trial utilized a 1:1:1 allocation ratio for the experimental and control groups. The sample size calculation formula, as implemented in PASS software, yielded a total of 333 cases, with 111 cases each in Groups A, B, and C. Accounting for a 10% dropout rate during the clinical trial, the actual enrolment target was set at 368 patients. The total number of subjects in each group is 123. In accordance with the established inclusion and exclusion criteria, a total of 331 patients were enrolled in this study and randomly assigned to Group A (*n* = 110), Group B (*n* = 110), and Group C (*n* = 111). During the course of the study, 16 patients elected to discontinue their participation: The distribution of patients across the three groups is as follows: 7 from Group A, 8 from Group B, and 1 from Group C. The study was completed and included in the statistical analysis by 315 patients across all three groups: 103 from Group A, 102 from Group B, and 110 from Group C (Figure 1).

### 2.4 Inclusion and exclusion criteria

The inclusion criteria were as follows: (i) age ≥ 18 years, either sex; (ii) laparoscopic radical surgery for CRC; (iii) NRS 2002 score ≥ 3 points (16); (iv) ability to take nutritional supplements orally; (v) no distant metastases detected on X-ray computed tomography (CT) scanning; (vi) American Society of Anesthesiologists (ASA) classification I–III (17); and (vii) fully accepted the study and signed a commitment form.

The exclusion criteria were as follows: (i) patients with intestinal or pyloric obstruction; (ii) reflux esophagitis, Los Angeles classification A or above; (iii) patients with gastroparesis; (iv) patients receiving enteral nutrition (EN) or parenteral nutrition (PN) supplementation due to severe malnutrition (≥9 points on the PG-SGA (Patient-Global Assessment)); (v) patients with comorbidities with underlying diseases of vital organs and hepatic or renal insufficiency; (vi) patients already enrolled in other clinical trials; (vii) patients or authorized persons unwilling to participate; and (viii) patients judged by the investigator to be unsuitable for participation and potential disputes.

### 2.5 Treatment protocol

Nutritional risk screening was conducted by a dietician prior to enrollment, with NRS 2002 scores ≥ 3 required for enrollment.

Anesthesia protocol: (i) Intravenous access was established, patients were connected to a monitor and received oxygen, and invasive arterial monitoring was performed under local anesthesia. Patients without contraindications were recommended to receive NSAIDs preoperatively and early postoperatively, not exceeding 3 days. (ii) Ultrasound-guided nerve blocks, such as erector spinae plane blocks, thoracic paravertebral blocks, and transversus abdominis plane blocks, were performed before surgery. (iii) Dexmedetomidine (0.3 μg/kg) was added to 100 mL of saline infused over 15 min; it was not used if the preoperative heart rate was lower than 50 beats/min or in cases of atrioventricular block. (iv) Induction of anesthesia: 0.03–0.05 mg/kg midazolam, 0.3–0.5 μg/kg sufentanil, 0.6–1 mg/kg rocuronium bromide, 0.1–0.15 mg/kg cisatracurium, 0.3 mg/kg etomidate (or 1–1.5 mg/kg propofol), followed by tracheal intubation after 5 min. (v) Generally, infusion of crystalloids at a rate of 1.5–2 mL/(kg·h) was sufficient to maintain fluid homeostasis for abdominal surgery. Zero fluid balance was advocated. In addition to appropriate fluid supplementation, small doses of vasoconstrictor drugs could be used to prevent excessive volume loading, postoperative tissue edema, and slow gastrointestinal recovery. (vi) Maintenance drugs: 2% sevoflurane or 6 mg/kg/h propofol; BIS monitoring was applied to maintain an appropriate depth of sedation (BIS value of 40–60); the drug was adjusted according to the patient’s blood pressure and heart rate and was discontinued during skin closure. At the end of surgery, intravenous nalbuphine 10 mg and tropisetron 5 mg could be administered. Bowel obstruction, preoperative neoadjuvant therapy, and the use of anti-anaerobic drugs can increase postoperative PONV, and the prophylactic use of two or three antiemetic drugs is recommended. (vii) The endotracheal tube was removed after the patient regained consciousness and had good independent breathing, after which the patient was sent to the PACU. (viii) Management of patients during their stay in the PACU was carried out according to the management system of the anesthesia recovery room of the Department of Anesthesiology. Postoperative analgesia: Intravenous patient-controlled analgesia: formula: 2–2.5 μg/kg sufentanil + 3 μg/kg dexmedetomidine + 40 mg nalbuphine + 15 mg tolansetron diluted in saline to 150 mL. The analgesic pump settings were as follows: background dose 3 mL/h, PCA bolus 3 mL, lockout time 20 min.

### 2.6 Surgical procedure

The criteria for radical surgery included the following: the distal and proximal margins of the colon tumor were at least 10 cm according to the CME principle, and the root needed to reach the D3 level; radical surgery for right hemicolectomy needed to be performed up to the left side of the superior mesenteric vein, with the vein skeletonized and the artery ligated at the root of its branch; radical surgery for transverse colon cancer needed to address the root of the middle colic artery; and radical surgery for left hemicolectomy needed to clear the left colic artery and the left branch of the middle colic artery (or the main trunk). Sigmoid colon cancer involved mainly the left colic vessels, sigmoid vessels, and superior rectal artery; the distal bowel should be preserved if it was more than 2 cm above the peritoneal reflection, and the superior rectal artery should be preserved. Radical treatment of rectal cancer was based on the principle of TME, and preservation of the left colic artery could be decided according to the situation; the proximal margin was 5 cm for upper, 2 cm for middle, and 1–2 cm for lower rectal cancer, ensuring a negative distal margin. Laparoscopic pressure was maintained at 11–14 mmHg; the number of ports was not specified; total laparoscopy (laparoscopic anastomosis), laparoscopic-assisted, or hand-assisted laparoscopy were available; anastomosis could be end-to-side, end-to-end (functional end-to-end), or side-to-side. At the end of the procedure, 1 drain was placed in the operative field. Surgical procedure: establishment of pneumoperitoneum, placement of trocars, exploration, positioning, mobilization, resection and anastomosis, reinforcement, closure of mesenteric defects, irrigation, placement of drains, and closure of the incision. Routine postoperative management included oxygen, cardiac monitoring, fluid replacement, and electrolytes (potassium chloride 4–6 g/day, sodium chloride 4.5 g/day); the dressing was changed every other day, and the drainage tube was removed after abdominal ultrasound was performed approximately 1–3 days postoperatively if drainage was minimal.

### 2.7 Nutrition interventions

(i) Preoperative oral CHOs level combined with postoperative EEN group (group A): patients were required to undergo NRS 2002 ≥ 3 nutritional risk screening for enrollment, 800 mL of oral CHOs (Oral CHOs nutritional solution contains 12.5 g of CHOs per 100 mL, comprising 80% maltodextrin and 20% crystalline sugar.) and fasting on the evening of the preoperative day, and 400 mL of oral CHOs (5 mL/kg) 2 h before the preoperative day. Enteral nutrition was given in the early postoperative period, and the specific enteral nutrition program was as follows: ① The day after surgery, patients should consume 20 mL of oral CHOs 6 h post-surgery until optimal tolerance is reached. Subsequently, the consumption of 50–100 mL of oral CHOs at three-hour intervals is recommended in order to achieve optimal recovery (total volume: 400 mL). ② On the 1st postoperative day, based on the patient’s tolerance, it is recommended to administer oral nutritional supplements (ONS) every 2 ~ 3 h (target total: 400–500 kcal) (Per 100 g, ONS provides 413.96 kcal of energy, with nutritional composition including: protein 15 g, fat 6.7 g, carbohydrates 72.3 g, sodium 300 mg, vitamin A 500 μg RE, vitamin D 1.2 μg, vitamin E 7.60 mg *α*-TE, vitamin B1 0.90 mg, vitamin B2 1.2 mg, vitamin B6 1.50 mg, vitamin B12 3.00 μg, vitamin C 100.0 mg, niacinamide 15.00 mg, folic acid 425 μg DFE, pantothenic acid 2.20 mg, calcium 80 mg, zinc 7.00 mg, taurine 110 mg). ONS every 2 ~ 3 h on the 1st day after surgery, depending on the patient’s tolerance. ③ Postoperative day 2: ONS every 2–3 h as tolerated by the patient. ONS targets a total of 750 kcal; ④ Postoperative day 3: ONS every 2–3 h as tolerated by the patient. ONS targets a total of 1,000 kcal, with the nutritionist following up and recording the other dietary energy/protein intake. ⑤ Postoperative day 4: ONS every 2–3 h according to the patient’s tolerance. ONS targets a total of 1,250 kcal, and nutritionist’s follow-up and record the energy/protein intake of other diets. ⑥ Postoperative days 5–7: adjust to semiliquid and gradually transition to soft food (target energy between 25–30 kcal/kg-d and protein between 1.0–1.2 g/kg-d).

(ii) Preoperative oral CHOs group (group B): Patients were screened for nutritional risk to ensure an NRS-2002 score ≥ 3. After enrollment, the patients were given 800 mL of oral CHOs on the evening before surgery and 400 mL of oral CHOs (5 mL/kg) 2 h before surgery. On the postoperative day, patients were given 20 mL of oral CHOs 6 h after surgery and 50–100 mL of oral CHOs every 3 h, depending on the patient’s tolerance (400 mL of oral CHOs in total). A liquid diet should be started 24 h after surgery under the guidance of the dietician and then transitioned from a liquid diet to a semiliquid diet or a liquid diet.

(iii) Conventional control group (Group C): Patients who are screened for nutritional risk and who meet the NRS-2002 criteria of ≥3 are included in this group. Patients follow the routine perioperative management of gastrointestinal surgery, and bowel preparation is carried out by adopting a semiliquid diet–fluid diet–fasting and rehydration before surgery. Patients were required to fast for 8 h before surgery, and enema cleansing was performed the night before surgery to ensure the smooth progress of the surgery. After postoperative evacuation, a liquid diet was started, and then a transition from a liquid diet to a semiliquid diet was made.

### 2.8 Data collection and outcomes

(i) General characterization: the following demographic and clinical data were obtained at admission: sex, age, height, weight, and past medical history. (ii) Laboratory test indicators: white blood cell count (WBC), red blood cell count (RBC), hemoglobin (HGB), platelet (PLT), total bilirubin (TBIL), alanine transaminase (ALT), aspartate aminotransferase (AST), total protein (TP), albumin (ALB), prealbumin (pALB), C-reactive protein (CRP), interleukin-6 (IL-6), and T lymphocyte subsets. (iii) Postoperative complications: pulmonary infections, abdominal infections, surgical incision infections, intestinal obstruction, anastomotic fistulae, and other complications. (iv) Postoperative recovery: time to first postoperative exhaust and defecation, total length of hospitalization, and postoperative length of stay.

### 2.9 Statistical analysis

Categorical variables are presented as counts with percentages, and numerical variables are presented as the means with standard deviations (SDs). Differences between groups were analyzed via Pearson’s chi-square test (*χ*^2^) or Fisher’s exact test, as appropriate for categorical variables; a t test was used for numerical variables. Statistical tests with a two-tailed *p*-value ≤ 0.05 were considered significant. The data were analyzed via IBM^®^ SPSS^®^ Statistics 23.0 (IBM, Chicago, IL, United States).

## 3 Results

### 3.1 Baseline characteristics of the subjects

As summarized in Table 1, 315 subjects (103 in group A, 102 in group B, and 110 in group C) were analyzed in this study. There were no significant differences in age, sex, height, weight, body mass index (BMI), systolic blood pressure (SBP), diastolic blood pressure (DBP), or comorbid underlying disease (e.g., hypertension, diabetes mellitus) related parameters at baseline (*p* > 0.05).

**Table 1:** Baseline characteristics of the participants.

Variables	Group A (103)	Group B (102)	Group C (110)	*P1*	*P2*	*P3*
Age, year	63.12 ± 10.19	64.21 ± 9.61	65.67 ± 8.87	0.417	0.559	0.328
Sex, *n* (%)				0.371	0.615	0.462
Male	40 (38.8)	45 (44.2)	51 (46.4)			
Female	63 (61.2)	57 (55.8)	59 (53.6)			
Height, cm	159.3 ± 7.54	157.6 ± 6.9	161.3 ± 5.80	0.712	0.180	0.315
Weight, kg	58.5 ± 7.33	56.8 ± 9.6	59.4 ± 6.84	0.812	0.313	0.273
BMI	22.01 ± 3.28	22.61 ± 3.02	22.08 ± 2.58	0.981	0.812	0.712
SBP	121.0 ± 15.8	128.9 ± 20.5	125.2 ± 15.4	0.890	1.120	0.112
DBP	73.1 ± 14.8	79.8 ± 15.1	79.0 ± 11.6	0.183	0.854	0.188
Comorbidity, *n* (%)						
Hypertension (*n*, %)	35.8%	29.2%	38.2%	0.662	0.694	1.130
Diabetes mellitus (*n*, %)	51%	47.92	51.5%	0.320	0.350	0.910

Data are presented as the means ± SDs or medians (IQRs) for continuous variables and *n* (%) for categorical variables. BMI, body mass index; SBP, systolic blood pressure; DBP, diastolic blood pressure. P1: Group A vs. Group B; P2: Group B vs. Group C; P3: Group A vs. Group C.

### 3.2 Preoperative immune status

As shown in Table 2, the immune status of the three groups of patients was further analyzed before surgery. Preoperative laboratory tests revealed no significant differences in immune-related indicators, including WBC, HGB, PLT, ALT, AST, TBIL, DBIL, TP, pALB, ALB, CRP, and IL-6; the percentage of T lymphocytes; the percentage of T helper/induced lymphocytes; the percentage of T suppressor cells; and the percentage of CD4+/CD8+ cells, among the three groups of patients (*p* > 0.05). These findings suggested that the three groups were comparable to each other.

**Table 2:** Comparison of various immunity indicators among the three groups of patients.

Variables	Group A (103)	Group B (102)	Group C (110)	*P1*	*P2*	*P3*
WBC (10*9/L)	6.03 ± 2.31	6.14 ± 1.61	6.28 ± 1.52	0.431	0.343	0.352
HGB (g/L)	116.2 ± 25.8	121.8 ± 30.1	115.4 ± 26.1	0.512	0.477	0.896
PLT (10*9/L)	224.1 ± 65.1	223.5 ± 87.1	234.2 ± 97.1	0.977	0.854	0.752
ALT (U/L)	17.1 ± 9.8	19.2 ± 10.5	16.8 ± 8.92	0.841	0.748	0.951
AST (U/L)	23.5 ± 12.8	23.7 ± 11.2	22.5 ± 3.91	0.916	0.613	0.792
TBIL (μmol/L)	13.1 ± 3.72	13.4 ± 5.72	13.5 ± 5.53	0.714	0.683	0.961
DBIL (μmol/L)	5.13 ± 3.13	4.38 ± 1.82	4.72 ± 2.84	0.876	0.881	0.792
TP (g/L)	62.2 ± 8.42	62.6 ± 7.91	63.4 ± 8.61	0.831	0.793	0.816
pALB (g/L)	224.7 ± 25.2	236.2 ± 28.2	224.1 ± 21.5	0.764	0.711	0.861
ALB (g/L)	42.8 ± 7.26	45.3 ± 7.17	46.2 ± 9.15	0.643	0.813	0.345
CRP	5.89 ± 4.57	5.65 ± 4.96	6.06 ± 5.85	0.151	0.184	0.261
IL-6	11.1 ± 21.3	12.47 ± 20.14	13.1 ± 21.4	0.564	0.712	0.815
T lymphocytes Percentage	65.4 ± 6.53	71.2 ± 6.91	65.2 ± 7.59	0.591	0.182	0.453
T-helper/inducer percentage	42.5 ± 12.3	40.5 ± 9.12	38.3 ± 8.12	0.891	0.561	0.442
T suppressor cell percentage	22.6 ± 5.72	24.6 ± 11.5	23.2 ± 6.25	0.229	0.276	0.642
CD4+/CD8+	1.86 ± 0.96	1.91 ± 1. 24	1.87 ± 0.84	0.761	0.854	0.766

Data are presented as the means ± SDs or medians (IQRs) for continuous variables and *n* (%) for categorical variables. WBC, white blood cell count; HGB, hemoglobin; PLT, platelet; ALT, alanine aminotransferase; AST, aspartate aminotransferase; TBIL, total bilirubin; TP, total protein; pALB, prealbumin; ALB, albumin; CRP, C-reactive protein; IL-6, interleukin-6. P1: Group A vs. Group B; P2: Group B vs. Group C; P3: Group A vs. Group C.

### 3.3 Postoperative nutrition-related serum biomarkers

The nutrition-related serum biomarkers related to the postoperative period in groups A, B and C are summarized in Table 3. TP has a shorter half-life and is less affected by acute stress, providing a more stable reflection of nutritional support efficacy. Therefore, we evaluated postoperative total protein levels in three groups of patients (18). Within 3 days after surgery, there was no significant difference in TP levels among groups A, B and C (*p* > 0.05), but at the one-week postoperative period, the TP levels of group A were significantly greater than those of groups B and C (*p* < 0.05). At either time point, there was no statistically significant difference in pALB or ALB among the three groups postoperatively (*p* > 0.05).

**Table 3:** Postoperative nutrition-related serum biomarkers indicators.

Variables	Postoperative time (t)	Group A (103)	Group B (102)	Group C (110)	*P1*	*P2*	*P3*
TP (g/L)	Day 1	57.8 ± 21.5	58.2 ± 19.5	58.8 ± 16.4	0.557	0.581	0.791
	Day 3	60.7 ± 9.12	57.3 ± 8.54	57.6 ± 8.37	0.334	0.851	0.411
	Day 7	61.5 ± 11.2	56.2 ± 8.36	55.4 ± 9.13	0.035*	0732	0.021*
pALB (g/L)	Day 1	204.4 ± 32.1	211.2 ± 29.5	204.5 ± 41.8	0.215	0.421	0.418
	Day 3	136.1 ± 52.4	138.3 ± 42.1	130.4 ± 42.6	0.612	0.416	0.316
	Day 7	96.8 ± 40.5	97.6 ± 40.2	98.5 ± 46.2	0.731	0.781	0.627
ALB (g/L)	Day 1	42.5 ± 4.17	43.8 ± 4.75	42.8 ± 5.41	0.612	0.785	0.821
	Day 3	37.5 ± 4.32	36.4 ± 4.31	35.9 ± 3.57	0.671	0.81	0.765
	Day 7	38.7 ± 8.15	35.7 ± 7.64	36.8 ± 7.16	0.151	0.783	0.246

Data are presented as the means ± SDs or medians (IQRs) for continuous variables and *n* (%) for categorical variables. TP, total protein; pALB, prealbumin; ALB, albumin. P1: Group A vs. Group B; P2: Group B vs. Group C; P3: Group A vs. Group C. *P-value statistically significant.

### 3.4 Comparison of postoperative hepatic and renal function recovery among the three groups of patients

Table 4 shows the postoperative liver and renal function indicators in groups A, B, and C. The results revealed no significant difference in ALT, AST, TBIL, DBIL, BUN or Cr among patients at 1, 3, and 7 days after surgery in among the preoperative oral CHOs combined with postoperative EEN group or the preoperative oral CHOs group or the conventional control group (*p* > 0.05).

**Table 4:** Postoperative liver and renal function indicators in patients.

Variables	Postoperative time (t)	Group A (103)	Group B (102)	Group C (110)	*P1*	*P2*	*P3*
ALT (U/L)	Day 1	17.2 ± 9.02	18.7 ± 12.1	16.7 ± 6.24	0.732	0.644	0.683
	Day 3	25.2 ± 10.2	27.7 ± 15.3	24.5 ± 18.2	0.561	0.461	0.672
	Day 7	30.4 ± 22.4	32.5 ± 25.4	29.4 ± 21.4	0.684	0.312	0.471
AST (U/L)	Day 1	23.9 ± 12.1	23.7 ± 10.1	22.4 ± 8.12	0.834	0.431	0.482
	Day 3	33.5 ± 48.3	35.1 ± 42.1	36.4 ± 47.6	0.413	0.816	0.416
	Day 7	33.1 ± 10.1	34.8 ± 16.2	35.3 ± 15.2	0.651	0.793	0.424
TBIL (μmol/L)	Day 1	13.5 ± 4.11	13.7 ± 5.71	13.8 ± 5.81	0.712	0.685	0.623
	Day 3	14.7 ± 5.01	15.8 ± 7.82	15.4 ± 6.63	0.706	0.674	0.573
	Day 7	13.2 ± 5.11	14.3 ± 8.12	13.4 ± 6.11	0.101	0.281	0.142
DBIL (μmol/L)	Day 1	5.12 ± 3.11	4.51 ± 1.84	4.86 ± 2.21	0.512	0.294	0.171
	Day 3	4.86 ± 2.62	5.14 ± 3.11	16.7 ± 6.24	0.732	0.644	0.683
	Day 7	17.2 ± 9.02	18.7 ± 12.1	16.7 ± 6.24	0.532	0.337	0.451
BUN (μmol/L)	Day 1	5.41 ± 5.12	4.55 ± 4.21	5.12 ± 2.43	0.332	0.214	0.883
	Day 3	4.52 ± 2.02	5.17 ± 2.15	6.17 ± 2.34	0.342	0.811	0.211
	Day 7	4.82 ± 2.52	5.84 ± 2.31	5.67 ± 2.18	0.251	0.812	0.317
Cr	Day 1	65.6 ± 11.3	71.2 ± 12.1	74.6 ± 20.4	0.228	0.878	0.415
	Day 3	62.2 ± 12.2	67.5 ± 11.5	68.9 ± 17.24	0.231	0.534	0.187
	Day 7	55.3 ± 11.2	59.7 ± 16.1	61.7 ± 21.4	0.252	0.713	0.181

Data are presented as the means ± SDs or medians (IQRs) for continuous variables and *n* (%) for categorical variables. ALT, alanine aminotransferase; AST, aspartate aminotransferase; TBIL, total bilirubin; DBIL, direct serum bilirubin; Cr, C-reactive protein; IL-6, interleukin-6. P1: Group A vs. Group B; P2: Group B vs. Group C; P3: Group A vs. Group C.

### 3.5 Postoperative inflammation indicators

Postoperative inflammatory and immune-related indicators are summarized in Table 5. The WBC, HGB and CRP levels of all patients were not significantly different among the three groups (*p* > 0.05). However, the decrease in the IL-6 indicator in group A was greater than that in groups B and C after the seventh postoperative day (*p* < 0.05). The percentage of T lymphocytes as a percentage of lymphocytes was much greater in group A than in groups B and C starting on the third postoperative day (*p* < 0.05), but there were no significant changes in the proportion of T-assisted/induced lymphocytes, the proportion of T-suppressed lymphocytes, or the proportion of CD4+/CD8+ lymphocytes (*p* > 0.05).

**Table 5:** Postoperative inflammatory indicators.

Variables	Postoperative time (t)	Group A (103)	Group B (102)	Group C (110)	*P1*	*P2*	*P3*
WBC (10*9/L)	Day 1	8.71 ± 2.02	9.21 ± 2.62	9.42 ± 2.95	0.535	0.814	0.481
	Day 3	6.52 ± 1.52	7.26 ± 2.14	6.13 ± 1.37	0.84	0.151	0.711
	Day 7	6.06 ± 2.14	6.51 ± 2.36	6.79 ± 2.13	0.604	0732	0.431
HGB (g/L)	Day 1	103.8 ± 22.1	107.2 ± 22.5	105.3 ± 22.8	0.614	0.911	0.817
	Day 3	105.2 ± 22.3	108.5 ± 22.1	105.8 ± 22.6	0.813	0.516	0.616
	Day 7	112.5 ± 19.1	109.4 ± 20.2	107.3 ± 21.2	0.621	0.691	0.326
CRP (mg/L)	Day 1	13.5 ± 4.11	13.7 ± 5.71	13.8 ± 5.81	0.712	0.685	0.623
	Day 3	41.2 ± 58.2	43.6 ± 48.7	45.8 ± 47.5	0.281	0.771	0.364
	Day 7	13.2 ± 5.11	14.3 ± 8.12	13.4 ± 6.11	0.101	0.281	0.142
IL-6 (pg/L)	Day 1	35.2 ± 3.11	32.1 ± 1.84	36.4 ± 2.21	0.512	0.294	0.171
	Day 3	28.7 ± 21.3	31.6 ± 23.1	30.8 ± 19.4	0.112	0.81	0.283
	Day 7	21.2 ± 20.8	26.7 ± 22.1	25.7 ± 6.24	0.032*	0.417	0.041*
T lymphocytes Percentage	Day 1	61.3 ± 7.15	59.5 ± 6.62	60.4 ± 7.95	0.535	0.814	0.781
	Day 3	67.5 ± 9.87	61.7 ± 9.15	62.8 ± 12.7	0.014*	0.581	0.021*
	Day 7	71.1 ± 6.25	63.9 ± 7.35	64.8 ± 6.64	0.011*	0.631	0.032*
T-helper/inducer percentage	Day 1	41.8 ± 12.6	37.2 ± 12.1	41.3 ± 13.1	0.315	0.421	0.916
	Day 3	40.3 ± 9.13	38.5 ± 10.1	39.5 ± 7.81	0.193	0.346	0.715
	Day 7	39.6 ± 8.21	37.4 ± 10.3	37.6 ± 11.3	0.782	0.224	0.226
T suppressor cell percentage	Day 1	23.5 ± 5.12	25.6 ± 4.72	23.8 ± 6.213	0.412	0.851	0.221
	Day 3	24.3 ± 8.45	24.6 ± 8.21	23.5 ± 7.53	0.731	0.472	0.372
	Day 7	23.2 ± 9.12	25.3 ± 8.12	24.4 ± 6.11	0.601	0.782	0.851
CD4+/CD8+	Day 1	1.82 ± 1.26	1.51 ± 0.84	1.86 ± 0.21	0.852	0.795	0.873
	Day 3	2.18 ± 1.62	1.89 ± 0.94	1.99 ± 0.84	0.725	0.681	0.874
	Day 7	1.87 ± 0.98	1.91 ± 1.15	1.72 ± 1.24	0.735	0.528	0.845

Data are presented as the means ± SDs or medians (IQRs) for continuous variables and *n* (%) for categorical variables. WBC, white blood cell count; HGB, hemoglobin; CRP, C-reactive protein; IL-6, interleukin-6. P1: Group A vs. Group B; P2: Group B vs. Group C; P3: Group A vs. Group C. *P-value statistically significant.

### 3.6 Postoperative intestinal functional status

The results revealed that the time to first defecation and exhaust was significantly earlier in group A than in groups B and C (*p* < 0.05) (Table 6).

**Table 6:** Postoperative intestinal function recovery indicators.

Variables	Group A (103)	Group B (102)	Group C (110)	*P1*	*P2*	*P3*
Time to first exhaust	1.69 ± 1.01	4.36	2.268 ± 0.89	0.031*	0.843	0.021*
Time to first defecation	2.26 ± 1.81	4.81 ± 1.62	3.46 ± 1.25	0.013*	0.379	0.023*

Data are presented as the means ± SDs or medians (IQRs) for continuous variables and *n* (%) for categorical variables. P1: Group A vs. Group B; P2: Group B vs. Group C; P3: Group A vs. Group C. *P-value statistically significant.

### 3.7 Postoperative intestinal functional status

Table 7 summarizes the postoperative complications, which mainly included lung infection, surgical incision infection, anastomotic fistula, abdominal infection, pulmonary embolism, and celiac leakage. The number of patients with all kinds of complications was small, and there was no significant difference between patients in groups A (0.078%), B (0.059%), and C (0.09%).

**Table 7:** Postoperative intestinal function recovery indicators.

Variables	Group A (103)	Group B (102)	Group C (110)	*P1*	*P2*	*P3*
Postoperative complications	8 (0.078%)	6 (0.059%)	10 (0.09%)	0.671	0.441	0.801
Lung infection	0	2	2			
Incision infections	2	0	2			
Anastomotic fistula	4	2	2			
Abdominal infection	0	0	4			
Pulmonary embolism	0	0	0			
Celiac fistula	2	2	0			
Total length of hospitalization	11.28 ± 2.41	13.65 ± 5.31	12.89 ± 6.43	0.453	0.694	0.836
Postoperative hospitalization days	8.69 ± 2.13	11.64 ± 4.16	11.68 ± 5.47	0.035*	0.895	0.031*

Data are presented as the means ± SDs or medians (IQRs) for continuous variables and *n* (%) for categorical variables. P1: Group A vs. Group B; P2: Group B vs. Group C; P3: Group A vs. Group C. *P-value statistically significant.

Furthermore, further analysis of the total length of hospital stays and postoperative length of stay in Groups A, B, and C revealed that there was no difference in the total number of days of hospitalization among the three groups, but the postoperative length of stay of patients in Group A (8.69 ± 2.13) was less than that in Groups B (11.64 ± 4.16) and C (11.68 ± 5.47) (*p* < 0.05).

### 3.8 Association between TP, IL-6, and T lymphocyte percentages and time to first exhaust or defecation

**Figure 2:**
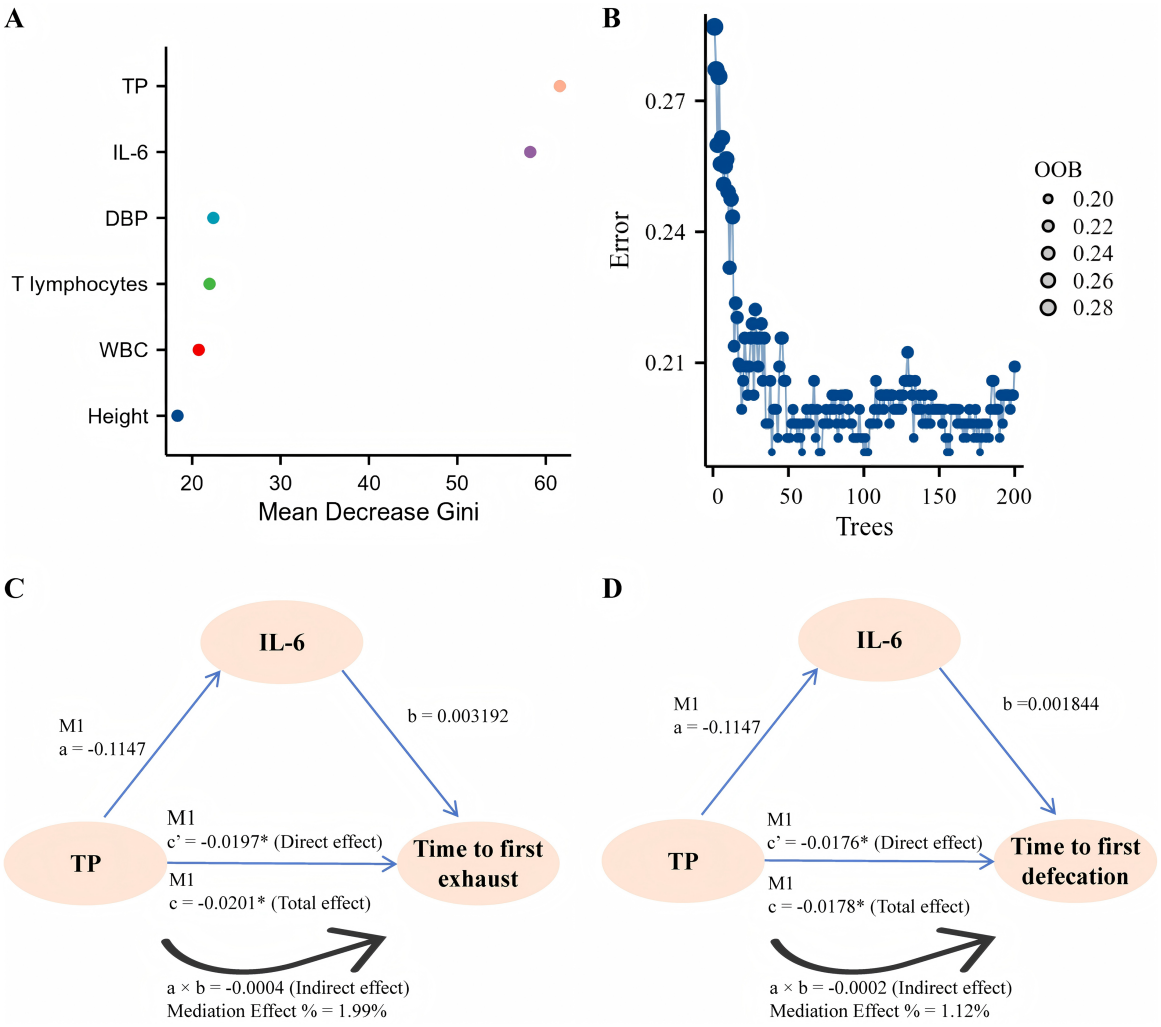
Correlation analysis. **(A)** The effect of each variable on the heterogeneity of observations at each node of the classification tree was calculated, with larger values indicating greater importance of the variable. **(B)** The number of decision trees is 200, and the OOB is visualized. **(C,D)** A graphic example of the mediation effect.

At baseline, the time to first defecation/exhaust was negatively associated with TP, T lymphocytes, and DBP but positively associated with IL-6, WBC, and height (Table 8). Random forest analysis of TP, IL-6, T lymphocytes, WBC, DBP, and height revealed that the predictor TP was the most important, followed by IL-6, and the out-of-bag (OOB) error rate was low, suggesting good predictive accuracy of the model (Figures 2A,B). Interestingly, further mediation effects analyses revealed the direct association of TP on postoperative day 7 with first defecation (total effect: −0.0201, direct effect: −0.0197)/time to defecation (total effect: −0.0178, direct effect: −0.0176), which was not observed in Groups B and C; moreover, the mediating effect of IL-6 was excluded (Table 9 and Figures 2C,D). The details of the testing procedures for the mediated effects model are summarized in Supplementary Table 1.

**Table 8:** Correlation analysis.

Variables	Time to first exhaust	Time to first defecation
TP	−0.362*	−0.251*
IL-6	0.211*	0.261*
Height	0.249*	0.257*
Weight	0.115	0.168
BMI	0.013	0.018
SBP	0.135	0.114
DBP	−0.214*	−0.198*
WBC	0.182*	0.198*
HGB	−0.015	0.139
PLT	0.071	0.066
ALT	0.094	0.049
AST	0.066	0.076
TBIL	0.004	−0.029
DBIL	0.064	−0.015
pALB	−0.083	−0.015
CRP	0.045	−0.072
T-helper/inducer percentage	−0.149	−0.022
T suppressor cell percentage	−0.136	−0.019
CD4+/CD8+	−0.048	0.180
T lymphocytes	−0.231*	−0.281*

BMI, body mass index; SBP, systolic blood pressure; DBP, diastolic blood pressure; WBC, white blood cell count; HGB, hemoglobin; PLT, platelet; ALT, alanine aminotransferase; AST, aspartate aminotransferase; TBIL, total bilirubin; TP, total protein; pALB, prealbumin; ALB, albumin; CRP, C-reactive protein; IL-6, interleukin-6. *P-value statistically significant.

**Table 9:** Summary of mediating effects.

Groups	Postoperative time (t)	Statistical term	Effect value	95% CI	*P*
Independent variable (TP)-mediating variable (IL-6)-dependent variable (time to first exhaust)					
A	Day 1	Total effect	0.0022	−0.0079 to 0.0119	0.6560
		ACME	0.0018	−0.0003 to 0.0045	0.1160
		ADE	0.0004	−0.0091 to 0.0101	0.9120
	Day 3	Total effect	0.0035	−0.0424 to 0.0501	0.9040
		ACME	−0.0041	−0.0163 to 0.0031	0.3640
		ADE	0.0076	−0.0403 to 0.0544	0.7920
	Day 7	Total effect	−0.0201	−0.0367 to −0.0028	0.0220*
		ACME	−0.0004	−0.0031 to 0.0015	0.7400
		ADE	−0.0197	−0.0360 to −0.0024	0.0260*
B	Day 1	Total effect	−0.0057	−0.0189 to 0.0077	0.3620
		ACME	−0.0003	−0.0033 to 0.0022	0.8300
		ADE	−0.0054	−0.0186 to 0.0080	0.3880
	Day 3	Total effect	−0.0422	−0.0422 to 0.0491	0.8460
		ACME	−0.0154	−0.0154 to 0.0027	0.2780
		ADE	−0.0359	−0.0359 to 0.0514	0.6800
	Day 7	Total effect	0.0032	−0.0189 to 0.0279	0.8060
		ACME	0.0009	−0.0026 to 0.0058	0.6500
		ADE	0.0024	−0.0208 to 0.0271	0.8520
C	Day 1	Total effect	−0.0011	−0.0087 to 0.0064	0.7460
		ACME	0.0000	−0.0019 to 0.0019	0.9920
		ADE	−0.0011	−0.0090 to 0.0067	0.7420
	Day 3	Total effect	0.0035	−0.0424 to 0.0501	0.9040
		ACME	−0.0041	−0.0163 to 0.0031	0.3640
		ADE	0.0076	−0.0403 to 0.0544	0.7920
	Day 7	Total effect	−0.0158	−0.0402 to 0.0101	0.1860
		ACME	−0.0015	−0.0015 to 0.0066	0.4160
		ADE	−0.0173	−0.0422 to 0.0096	0.1660
Independent variable (TP)-mediating variable (IL-6)-dependent variable (time to first defecation)					
A	Day 1	Total effect	−0.0005	−0.0167 to 0.0153	0.9180
		ACME	−0.0002	−0.0042 to 0.0034	0.9720
		ADE	−0.0003	−0.0174 to −0.0162	0.9480
	Day 3	Total effect	−0.0329	−0.1074 to 0.0441	0.4160
		ACME	0.0032	−0.0098 to 0.0169	0.6300
		ADE	−0.0361	−0.1145 to 0.0417	0.3900
	Day 7	Total effect	−0.0178	−0.0331 to −0.004	0.0440*
		ACME	−0.0002	−0.0027 to 0.0014	0.8160
		ADE	−0.0197	−0.0330 to −0.0003	0.0420*
B	Day 1	Total effect	−0.0062	−0.0228 to 0.0093	0.4560
		ACME	−0.0035	−0.0088 to −0.0001	0.0420*
		ADE	−0.0027	−0.0190 to 0.0118	0.7880
	Day 3	Total effect	−0.0390	−0.1010 to 0.0318	0.2500
		ACME	0.0036	−0.0098 to 0.0191	0.6040
		ADE	−0.0426	−01028 to 0.0301	0.2040
	Day 7	Total effect	0.0195	−0.0067 to 0.0486	0.1540
		ACME	−0.0004	−0.0038 to 0.0032	0.9180
		ADE	0.0198	−0.0062 to 0.0487	0.1580
C	Day 1	Total effect	0.0010	−0.0116 to 0.0137	0.8780
		ACME	−0.0000	−0.0027 to 0.0025	0.9400
		ADE	0.0011	−0.0116 to 0.0140	0.8840
	Day3	Total effect	0.0234	−0.0357 to 0.0738	0.4680
		ACME	−0.0013	−0.0127 to 0.0066	0.7180
		ADE	0.0247	−0.0331 to 0.0778	0.4400
	Day 7	Total effect	−0.0022	−0.0227 to0.0178	0.8540
		ACME	−0.0005	−0.0036 to 0.0020	0.8020
		ADE	−0.0017	−0.0226 to 0.01882	0.8840

ACME, average causal mediation effect; ADE, average direct effect; 95% CI, confidence interval; TP, total protein; IL-6, interleukin 6. *P-value statistically significant.

Effect value: regression coefficient.

Independent variable: TP.

Dependent variable: time to first exhaust/defecation.

Mediating variable: IL-6.

### 3.9 Total protein status prediction time to first exhaust/defecation

To predict the time to first exhaust/defecation, hierarchical linear regression analysis was performed (Table 10). Hierarchical linear regression models revealed that TP combined with IL-6, WBC count, height, DBP, and T lymphocytes explained 29.7% of the time to first exhaust values and 30.7% of the time to defecate values.

**Table 10:** Hierarchical regression analysis of first exhaust/defecation as the dependent variable (*n* = 315).

Hierarchical regression								
Step 1				Step 2				
	*β*	VIF	*t*	*P*	*β*	VIF	*t*	*P*
Dependent variable: time to first exhaust								
Constant	7.7	2.756	2.807	0.006**	8.603	2.814	3.057	0.003**
T lymphocytes	−0.118	1.117	−1.081	0.042*	−0.120	1.317	−1.179	0.041*
Height	−0.024	1.615	−1.631	0.106	−0.024	1.815	−1.628	0.107
DBP	−0.012	1.257	−1.758	0.082	−0.013	1.527	−1.849	0.068
WBC	−0.029	2.246	0.636	0.526	0.035	2.546	0.765	0.446
IL-6	−0.201	2.155	−2.036	0.037*	−0.231	2.315	−2.227	0.029*
TP					−0.253	1.419	−2.380	0.021*
*R* ^2^	0.279				0.297			
Adjust *R*^2^	0.230				0.239			
*F*	*F* = 2.604, *p =* 0.047*				*F* = 2.667, *p* = 0.038*			
Dependent variable: time to first defecation								
Constant	5.864	4.787	1.225	0.004**	7.617	4.858	−1.568	0.002**
T lymphocytes	−0.122	1.329	−1.778	0.039*	−0.128	1.429	−1.886	0.034*
Height	0.061	1.726	2.405	0.018*	0.061	1.825	2.400	0.017*
DBP	0.016	1.378	−0.547	0.586	−0.057	1.478	−0.732	0.466
WBC	−0.043	2.412	1.311	0.193	0.017	2.532	0.141	0.153
IL-6	−0.202	2.208	−2.225	0.022*	−0.231	2.358	−2.143	0.017*
TP					−0.271	1.516	−2.668	0.019*
*R* ^2^	0.279				0.307			
Adjust *R*^2^	0.230				0.250			
*F*	*F* = 2.686, *p =* 0.045*				*F* = 2.894, *p =* 0.019*			

DBP, diastolic blood pressure; WBC, white blood cell; TP, total protein; IL-6, interleukin-6. *p-value statistically significant, **p-value less than 0.01.

## 4 Discussion

The present data demonstrated that on postoperative day seven, patients in the group with preoperative oral CHO combined with postoperative ENN (Group A) had significantly greater TP levels and a faster reduction in the level of the inflammatory indicator IL-6 than did those in the preoperative oral CHO group (Group B) and the conventional control group (Group C). Additionally, patients in Group A had a shorter time to first postoperative exhaust/defecation and shorter postoperative hospitalization than did those in Groups B and C. Importantly, TP levels combined with IL-6, WBC, height, DBP, and T lymphocytes could be predictors of the time to first exhaust/defecation. To the best of our knowledge, this is the first study to investigate the effects of preoperative oral CHO combined with postoperative ENN on the perioperative recovery of CRC patients. These findings could provide a novel nutritional strategy for CRC patients in the perioperative period and accelerate their postoperative recovery.

Patients with CRC are usually asked to abstain from food and drink for 1 day prior to surgery and to take laxatives and perform cleansing enemas to reduce possible intraoperative complications (e.g., anesthetic aspiration, anastomotic fistula, and incisional infection) (19). However, prolonged dietary abstinence depletes hepatic glycogen reserves and increases the risk of causing insulin resistance (20). Consequently, preoperative oral CHO nutritional regimens are routinely used to prevent or ameliorate postoperative insulin resistance, hyperkalemia, and inflammatory responses in patients with CRC (21). Increasing evidence suggests that postoperative ENN also promotes postoperative bowel motility and increases TP and transferrin levels in patients with gastrointestinal tumors (22). Although, in this study, no differences were observed between preoperative oral CHO and conventional controls, we found that preoperative oral CHO combined with postoperative ENN significantly elevated patients’ postoperative TP levels compared with those in the conventional control and preoperative oral CHO groups. TP levels are critical to patients’ postoperative recovery (23). Clinical studies have shown that, compared with fasting patients, patients in the EEN group had significantly greater total protein levels on both the third and seventh postoperative days and had shorter periods of postoperative fever and flatus (24). Multiple meta-analyses have also demonstrated that patients with high total serum protein levels have a shorter recovery time from postoperative bowel function and fewer complications than patients with low total protein levels do (25–27). The findings of this study suggested that a strategy integrating preoperative oral carbohydrate administration with postoperative ENN effectively enhanced postoperative total protein and albumin levels. Although albumin and TP serve as key indicators reflecting acute inflammation and stress, maintaining and elevating their levels is clinically regarded as a positive signal of effective nutritional support and alleviated metabolic stress, and is associated with improved clinical outcomes (28–30). Consequently, it can be hypothesized that the intervention may have contributed to the recovery of these indicators by concurrently alleviating surgical stress and providing timely nutritional support.

Our study also revealed a significant decrease in IL-6 levels in the preoperative oral CHO combined with postoperative ENN group compared with the preoperative oral CHO group and the conventional control group. This finding is consistent with the results of recent RCTs for patients undergoing surgery for esophageal (31) and gastric cancer (32). Numerous clinical studies have shown that ENN exerts immunomodulatory and anti-inflammatory effects. A study of postoperative ENN intervention in patients with gastric cancer revealed that the serum levels of IL-6 and tumor necrosis factor-alpha (TNF-α) were significantly lower in the intervention group than in the control group, resulting in a lower degree of inflammation (33). Similar intervention outcomes were observed in patients with colitis, with EEN reducing serum IL-6, interleukin 1 (IL-1), and TNF-α levels and attenuating the state of intestinal oxidative stress (34). Moreover, we found that patients in the preoperative oral CHO combined with postoperative ENN group had a greater percentage of T lymphocytes to lymphocytes than did those in the preoperative oral CHO group and the conventional control group. These findings indicate that preoperative oral CHO combined with postoperative ENN may promote the recovery of postoperative immune function in CRC patients.

The recovery of bowel function in colon cancer patients after surgery is mainly indicated by the time to exhaust and defecation (35, 36). Numerous RCTs have demonstrated that postoperative ENN promotes intestinal peristalsis, prevents intestinal mucosal injury, and facilitates the absorption of nutritional preparations. A study of a postoperative ENN intervention that included patients with pancreatic, esophageal, and gastric cancers revealed that the ENN intervention upregulated patients’ serum TP levels and that patients in the ENN group had shorter times to first exhaust and defecation than did those in the control group (37). Similarly, in this study, patients in the preoperative oral CHO combined with postoperative ENN group had a shorter time to first exhaust/defecation than did those in the preoperative oral CHO group and the conventional control group.

Mechanistic studies support the concept that protein supplementation or increasing total protein levels could shorten the time to first postoperative exhaustion/defecation by inhibiting inflammatory markers [CRP (38), IL-6 (39), and IL-10 (40)] or signaling pathways [PI3K/AKT (41) and NF-κB (42)]. A prospective cohort study revealed that TP levels were negatively associated with length of hospitalization and time to first defecation (43). Additionally, IL-4, IL-6, IL-10, and TNF-α levels are positively associated with the time to first exhaust/defecation (44). In our study, patients in the preoperative oral CHO combined with postoperative ENN group had less postoperative hospitalization than did those in the preoperative oral CHO group and the conventional control group. Furthermore, total protein, T lymphocytes and DBP were negatively associated with the time to first exhaust/defecation, whereas IL-6, height and WBC count were positively associated with the time to first exhaust/defecation. Random forest analysis revealed that TP was the most important predictor, followed by IL-6. Random forest analysis revealed that TP was the most important predictor, followed by IL-6. Lactoferrin supplementation has been shown to exert an anti-inflammatory effect by reducing circulating IL-6 and TNF-α levels (45). However, in our study, mediated effect analysis revealed that TP on postoperative day 7 was directly associated with the time to first exhaust/defecation, excluding the mediating effect of IL-6. Therefore, on the basis of the above studies, we concluded that preoperative oral CHO combined with postoperative ENN increased TP levels in postoperative patients and that TP was associated with the time to first exhaust/defecation.

Recently, a machine learning-based prediction model revealed that time to first postoperative feeding, age, probiotics, and oral antibiotics for bowel preparation were independent risk factors for time to first defecation in CRC patients (46). A predictive model for hospitalization time after gastric cancer surgery revealed that four clinical characteristics, the neutrophil–lymphocyte ratio (NLR) on postoperative day one, the NLR on postoperative day three, the preoperative prognostic nutrient index and the first anal exhaust, were good predictors of hospitalization time (47). Studies on postoperative ENN interventions [intestinal anastomosis in pediatrics (48), radical total gastrectomy for gastric cancer (49), intestinal obstruction (50), etc.] have shown that ENN could contribute to the recovery of postoperative bowel function. However, few studies have delved into multivariate analysis of the time to first postoperative exhaust/defecation for patients with CRC. The present study demonstrated that TP combined with IL-6, WBC, height, DBP and T lymphocytes can be used as predictors of the time to first exhaust/defecation.

In this study, we investigated the value of preoperative oral CHO combined with postoperative ENN in CRC patients. The results of the present study revealed that preoperative oral CHO combined with postoperative ENN among CRC patients could significantly optimized postoperative nutrition-related indicators, strengthen immune function, promote early recovery of intestinal function, and significantly shorten the duration of postoperative hospitalization. It is important to note that this study utilized serum TP and albumin as the primary nutrition-related indicators. It is acknowledged that in circumstances involving stress, such as surgical procedures, the levels of these visceral proteins are considerably impacted by the systemic inflammatory response syndrome. The reduced concentrations observed may be indicative of heightened catabolism and modified capillary permeability, rather than being solely attributable to inadequate nutritional intake (51–53). Consequently, it is recommended that they be regarded more accurately as composite markers of nutritional risk and inflammatory stress. Notwithstanding this limitation, patients in the intervention group exhibited elevated protein levels and enhanced clinical outcomes (e.g., reduced hospitalization durations), providing substantial evidence that our integrated nutritional strategy effectively mitigated metabolic disturbances and catabolic states induced by surgical trauma. It is recommended that future studies incorporate more specific nutritional assessment tools (e.g., body composition analysis, grip strength) in order to provide more comprehensive evidence. Meanwhile, there were several limitations in this study. Firstly, long-term prognostic data were not obtained, and more experimental data are needed to validate whether EEN support impacts patient prognosis. Secondly, potential confounders were not adequately controlled, and some clinical variables (e.g., tumor stage and differences in drug combinations) were not adequately matched, which may affect the interpretation of the results. Moreover, this study did not assess patients’ postoperative quality of life (e.g., physical recovery, psychological status) and focused only on physiologic indicators, which failed to fully reflect the clinical value of the intervention. Therefore, we anticipate that further studies will be conducted to clarify the relationships among these variables.

## Data Availability

The original contributions presented in the study are included in the article/Supplementary material, further inquiries can be directed to the corresponding author.
